# Exploring the Prevalence of Oral Features for Early Detection of PTEN Hamartoma Tumour Syndrome

**DOI:** 10.1016/j.identj.2024.04.014

**Published:** 2024-05-01

**Authors:** Ane J. Schei-Andersen, Bart van Oirschot, Meggie M.C.M. Drissen, Jolanda Schieving, Janneke H.M. Schuurs-Hoeijmakers, Janet R. Vos, Claire M. Barton, Nicoline Hoogerbrugge

**Affiliations:** a Department of Human Genetics, Radboud University Medical Center, Nijmegen, the Netherlands; b Department of Dentistry, Radboud University Medical Center, Nijmegen, the Netherlands; c Department of Paediatric Neurology, Radboud University Medical Center, Nijmegen, the Netherlands; d Radboud Institute for Medical Innovation, Radboud University Medical Center, Nijmegen, the Netherlands; e PTEN Research Foundation, Registered office: 4th Floor, St James House, St James Square, Cheltenham, UK; f Barton Oncology Ltd, Dormers, The Green, Croxley Green, Hertfordshire, UK

**Keywords:** PHTS, PTEN, Cowden syndrome, Recognition, Prevention, Phenotype

## Abstract

**Aims:**

Patients with PTEN hamartoma tumour syndrome (PHTS) have an increased risk of developing cancer due to a pathogenic germline variant in the *PTEN* tumour suppressor gene. Early recognition of PHTS facilitates initiation of cancer surveillance which is highly effective in preventing the development of advanced malignancies. PHTS is rare and due to its varied phenotype, even within families, oral abnormalities may be a valuable tool in the identification of these patients at an early stage before cancer development.

**Materials and methods:**

Between 1997 and 2020, phenotypic characteristics were evaluated in 81 paediatric (median age: 9 years) and 86 adult (median age: 40 years) PHTS patients by one of 2 medical experts during yearly surveillance visits at a Dutch PHTS expertise centre. Oral features evaluated included gingival hypertrophy, oral papillomas, and high palate (in adults).

**Results:**

Within adults, gingival hypertrophy was present in 94%, oral papillomas in 88%, and a high palate in 89%. All adult patients had at least one of these oral features, and 99% showed at least 2 oral features. Oral features were less common in paediatric patients, especially under 11 years of age. Gingival hypertrophy was observed in 44% and oral papillomas in 54% of paediatric patients.

**Conclusions:**

The presence of 2 or 3 oral features may indicate PHTS in adults or adolescents, especially if macrocephaly is present. Dental professionals are well-positioned to recognise these oral manifestations could be related to PHTS. They can initiate an overall clinical assessment of the patient by alerting the patient's medical practitioner of the findings and the possible need for genetic testing. This could significantly improve outcomes, including life expectancy, for patients and possibly for their relatives.

**Clinical relevance:**

Dental professionals are ideally placed to recognise oral features and initiate early assessment of PHTS which could significantly improve patient outcomes.

## Introduction

PTEN hamartoma tumour syndrome (PHTS) is a rare hereditary condition caused by a loss-of-function germline variant in the phosphatase and tensin homolog (*PTEN*) gene. This tumour suppressor gene encodes a lipid phosphatase which is a negative regulator of the phosphatidylinositol 3-kinase pathway. These variants may be inherited in a family for generations, following an autosomal dominant pattern. However, 10% to 45% of cases are due to new (*de novo*) mutations.^1^

Clinical manifestations of PHTS are diverse and include autism spectrum disorder, developmental delay, macrocephaly, benign hamartomas and soft tissue overgrowth, vascular malformations, immune dysfunction, as well as an increased hereditary risk of malignancy (particularly breast, thyroid, and endometrial cancer).^2^, ^3^, ^4^ Malignancies often occur at a relatively young age.^5^^,^^6^ Symptoms of PHTS vary in incidence and severity, even within a family with the same germline variant.

Determining the prevalence of PHTS is complex due to the varied presentations and diagnoses and because some features, such as breast fibroadenomas and thyroid nodules, also commonly occur in the general population.^7^ Overall, it is estimated that about 1 in 200,000 individuals have PHTS (corresponding to around 47,000 individuals worldwide), although this is likely an underestimate. Amongst individuals with autism spectrum disorder, around 2% are thought to have PHTS (17% if macrocephaly is also present).^8^

A comprehensive cancer surveillance programme is recommended for all PHTS patients.^9^^,^^10^ Cancer surveillance has proved to be highly effective in preventing advanced malignancy in PHTS patients.^11^, ^12^, ^13^, ^14^ However, initiation of surveillance requires timely recognition. Unfortunately, a common problem with hereditary cancer syndromes is poor recognition of patients at risk, leading to underdiagnosis and delayed diagnosis.^15^^,^^16^ As a result, it is likely that a substantial proportion of PHTS patients remain undiagnosed and therefore unaware of their risk and miss the opportunity for surveillance.

Paediatric PHTS patients can present with macrocephaly, autism spectrum disorder or developmental delay which is more likely to trigger genetic testing.^4^ However, for those without autism or developmental delay (a substantial proportion of individuals with PHTS), there are no obvious “red flags” and PHTS may not be considered until the first or even second cancer is diagnosed. A strategy to identify children for genetic testing based on computerised searches of clinical billing and diagnostic codes was successful in a pilot study, but is not yet in routine use.^17^ No such strategy has been developed yet for adults.

Many case reports indicate that oral abnormalities, notably oral papillomas, generalised gingival hypertrophy and a high arched palate, may be common in adults with PHTS.^18^, ^19^, ^20^, ^21^, ^22^, ^23^ Furthermore, in mouse models, selective *PTEN* deletion in epithelial cells leads to the development of oral mucosal papillomas.^24^ Overall, the most common clinical features of PHTS are oral signs (e.g. oral papillomas, generalised gingival hypertrophy and high arched palate), macrocephaly (>66% of patients) and an increased lifetime risk of cancer (>85% of patients).^5^^,^^25^ These patients often receive repeated advice and encouragement from dental professionals to improve their oral hygiene and to reduce the gingival tissue, although this is actually caused by their condition and not due to negligence.

This paper is based on the previously published evaluation of oral features in combination with multinodular goitre and macrocephaly.^25^ In this paper, the objective was to describe and to investigate the frequency and sensitivity of oral features in PHTS patients. Dental practitioners are ideally placed to detect oral features of PHTS in their patients – before cancer development – and could play a key role in earlier recognition.

## Material and methods

### Setting

This was a retrospective cohort study including PHTS patients under surveillance at the Radboud University Medical Center. Patients with a genetically confirmed diagnosis of PHTS were selected using standard genetic criteria.^26^ The phenotype of paediatric (<18 years) and adult (≥18 years) patients was evaluated by one of 2 medical experts (JS or NH) during yearly surveillance visits between 1997 and 2020.

### Phenotypic information

During surveillance, paediatric and adult patients were examined for gingival hypertrophy and oral papillomas by visual inspection. The presence of a high palate (defined as a palatal height more than twice the height of the teeth at the level of the first permanent molar) was also evaluated but only in adults. Palatal height could not be reliably assessed in children due to age-related variability, difficulty of measurement, mouth breathing or excessive thumb sucking.^27^^,^^28^

Data on phenotypic characteristics were extracted for analysis from the patients’ medical records. The presence of each characteristic was assessed up until the last day of clinical follow-up. If conflicting reports on a characteristic were found, the most recent data was utilised. Based on the prevalence of the characteristics in different age groups, combinations of characteristics were defined and evaluated for their potential to identify adult patients with PHTS. Photographs of oral characteristics were taken with informed consent from patients.

### Statistical analyses

Continuous data were reported as median and interquartile range (IQR), and categorical variables as counts and percentages. The prevalence and sensitivity of phenotypic characteristics was assessed for the entire cohort and for paediatric and adult patients separately. Differences between paediatric and adult patients were assessed using the chi-squared or Fisher's exact test.

Differences in sensitivity between combinations of characteristics were assessed using the McNemar's exact test in patients with complete data for both combinations. All statistical analyses were performed in R version 3.6.2 and 2-sided *P*-values <.05 were considered statistically significant.

Data from non-PHTS patients and patients with other syndromes were not collected in this study. Hence the specificity and predictive value could not be assessed. Instead, published data on the prevalence of oral papillomas, gingival hypertrophy and high palate was used to estimate specificity for the 3 oral features.

## Results

### Study participants

In total, 167 PHTS patients were included in this study, of whom 81 (49%) were paediatric and 86 (51%) were adults. The median age at last day of follow-up for the entire cohort was 25 years (IQR: 9-41 years). For paediatric patients, median age was 9 years (IQR: 5-12 years), and 40 years (IQR: 28-49 years) for adults. The cohort included 88 females (53%), of whom 29 were paediatric.

Of all adult patients, 41 (48%) were index cases (the first to be diagnosed within a family) and 45 (52%) were non-index. About half of the non-index cases were parents of an index case, and the other half were children, siblings, and second-degree relatives of an index case, in approximately equal proportions.

### Prevalence and sensitivity of oral features

**Figure 1:**
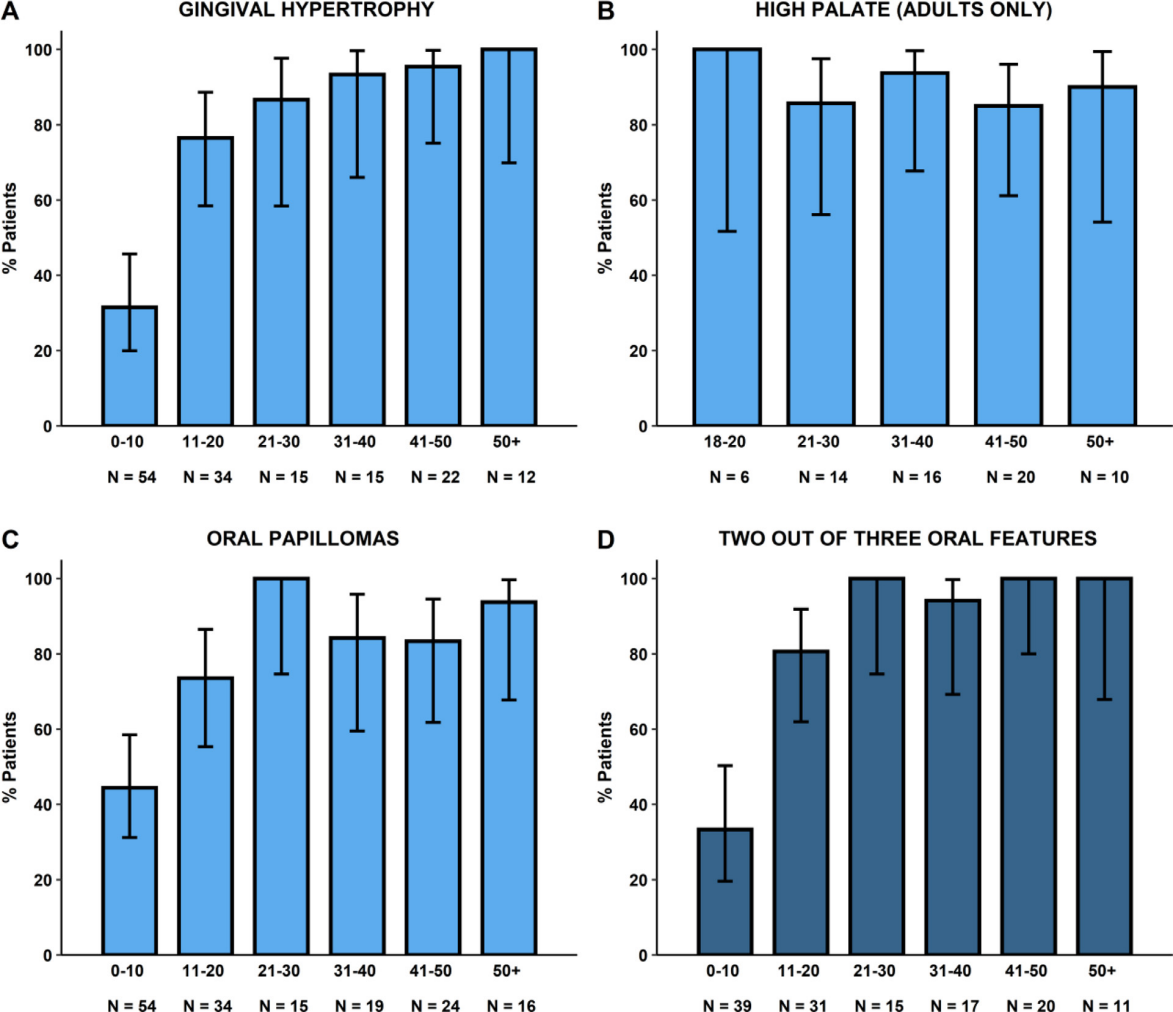
Age distribution of oral features in PHTS patients. (A) Gingival hypertrophy, (B) high palate (adults only), (C) oral papillomas, and (D) ≥2 out of 3 oral features.

**Figure 2:**
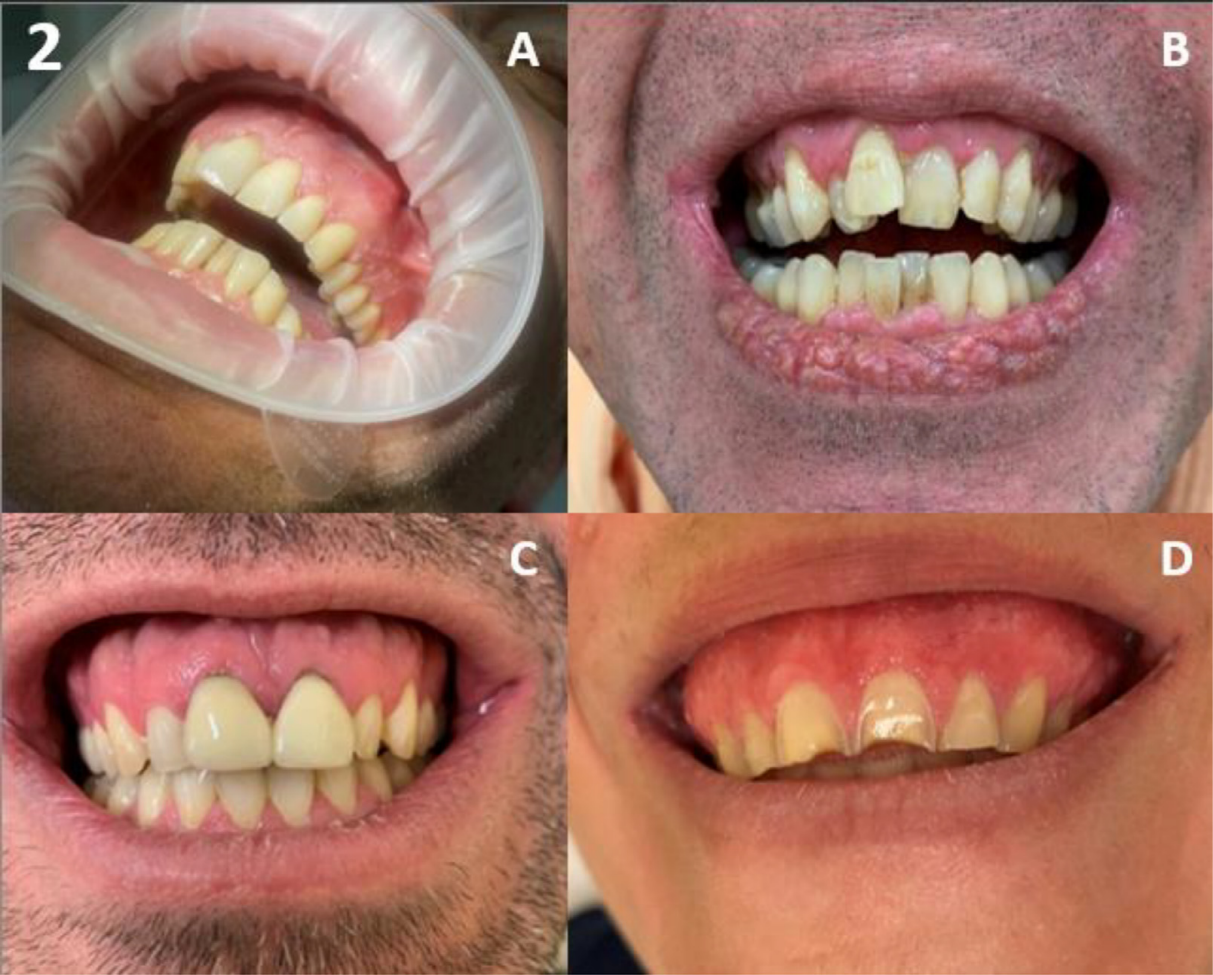
Gingival hypertrophy. (A-D) Hypertrophy of gingival tissue in PHTS patients. In (B), papilloma of the lip is also present and in (D) papillomatosis of the gingival tissue is present.

**Figure 3:**
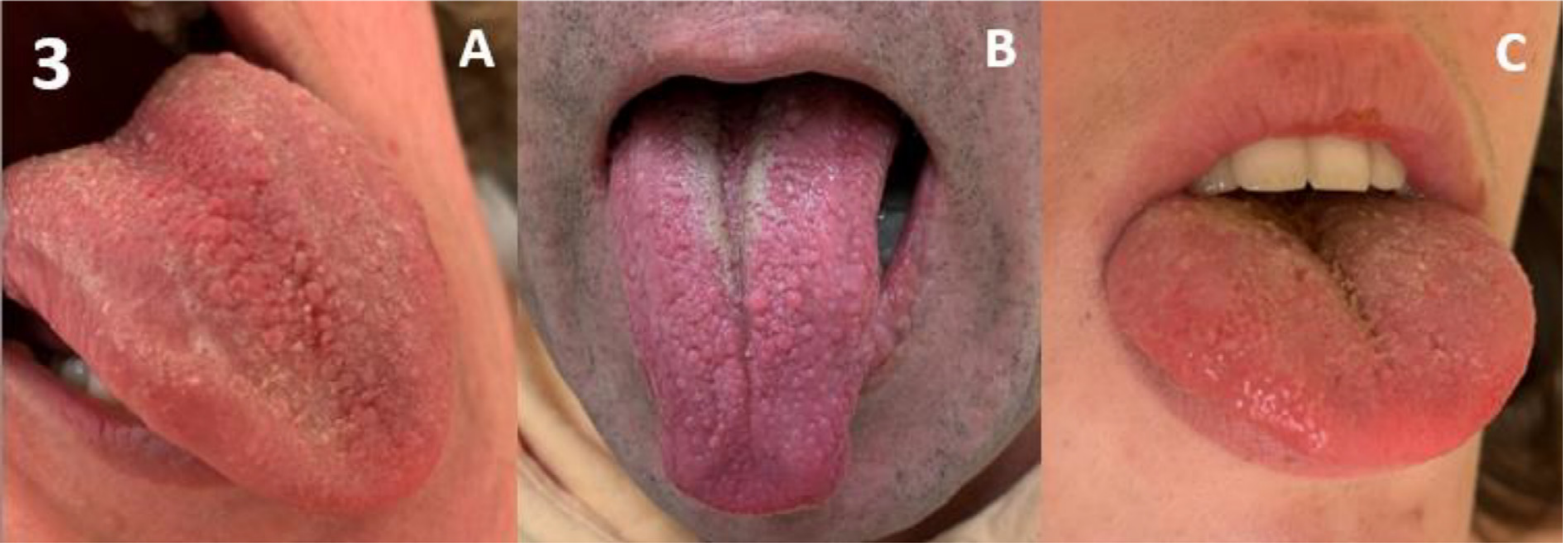
Papillary tongue. (A-C) Papillary tongue: papillomas of the tongue affecting the entire dorsal surface to the line of circumvallate papillae.

**Figure 4:**
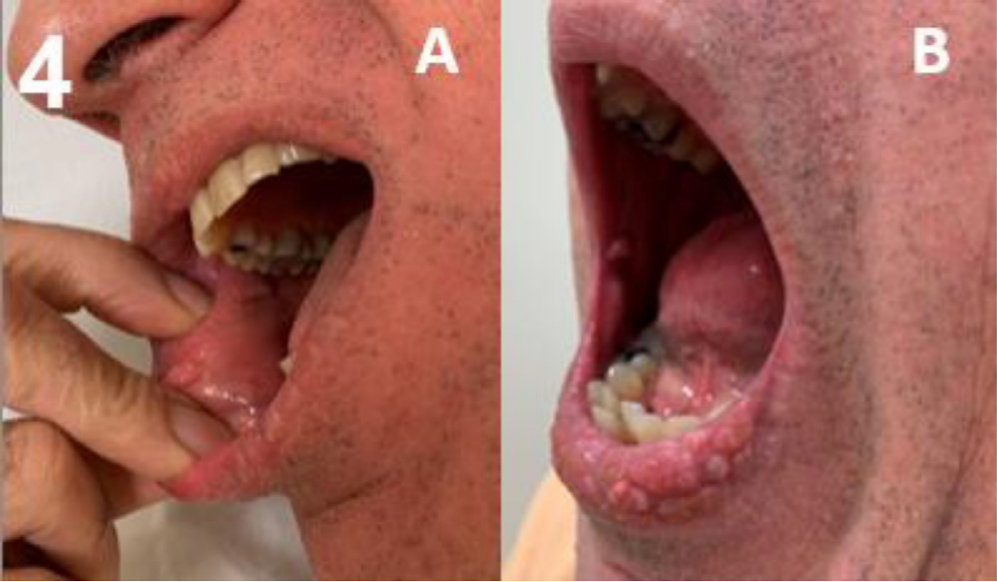
Papillary buccal mucosa. Papillomas of the buccal mucosa.

**Figure 5:**
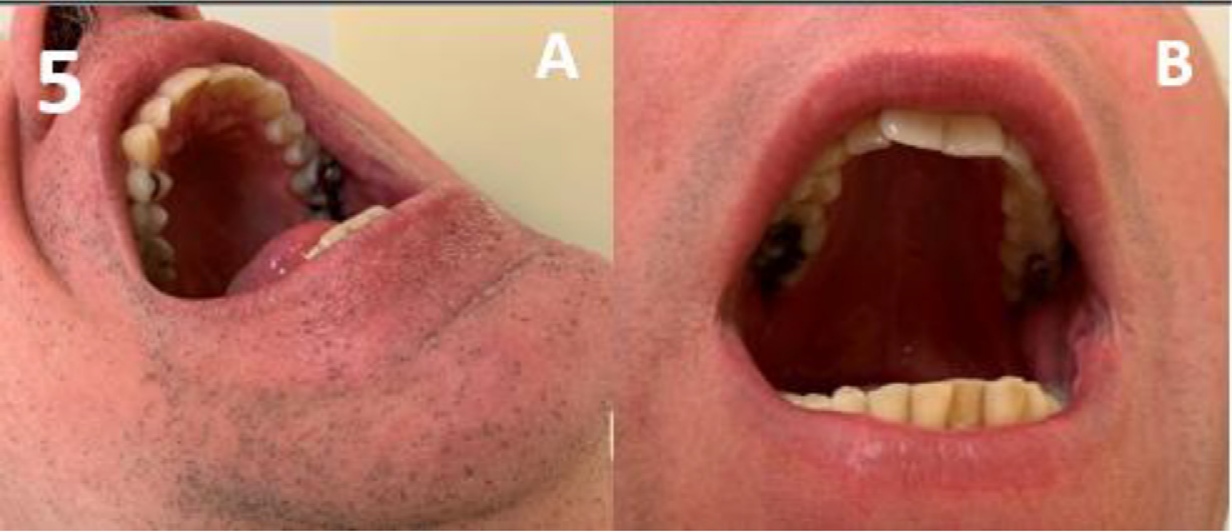
High palate, here defined as a palatal height twice the height of the first permanent molar.

Amongst the 167 patients, the majority had oral features, including generalised gingival hypertrophy in 68%, and oral papillomas in 71% (67% tongue papillomas & 31% mucosal papillomas) (Table). A high palate was found in 89% of adults. Gingival hypertrophy and oral papillomas gradually developed with increasing age (Figure 1). Overall, gingival hypertrophy was observed in 44% of paediatric patients and 94% of adults, and oral papillomas were observed in 54% and 88%, respectively. In both paediatric and adult patients, papillomas were located more often on the tongue (54% of paediatric patients and 80% of adults) compared to the mucosa (0% of paediatric patients and 62% of adults). Typical examples of the 3 oral features are shown in Figure 2, Figure 3, Figure 4 to 5. Note that papillomas affecting the lips are also apparent in some of these patients (Figures 2B and 4B).^7^

**Table:** Prevalence and sensitivity of (combined) oral features in PHTS patients^25^

	All (N = 167)		<18 (N = 81)		≥18 (N = 86)	
	n/N	% (95% CI)	n/N	% (95% CI)	n/N	% (95% CI)
***Single oral features***						
Gingival hypertrophy	103/152	68% (60%-75%)	36/81	44% (34%-56%)	67/71	94% (85%-98%)
High palate	59/66	89% (79%-95%)	NA	NA	59/66	89% (79%-95%)
Oral papillomas	115/162	71% (63%-78%)	44/81	54% (43%-65%)	71/81	88% (78%-94%)
Tongue papillomas	109/162	67% (59%-74%)	44/81	54% (43%-65%)	65/81	80% (70%-88%)
Mucosal papillomas	50/162	31% (24%-39%)	0/81	0% (0%-6%)	50/81	62% (50%-72%)
Combinations of oral features*						
≥ 1 oral feature	131/131	100% (96%-100%)	49/49	100% (91%-100%)	82/82	100% (94%-100%)
≥ 2 oral features	100/133	75% (67%-82%)	31/63	49% (37%-62%)	69/70	99% (91%-100%)
All 3 oral features	NA	NA	NA	NA	46/66	70% (57%-80%)

CI, confidence interval; n, number of patients with the feature; N, total number of patients in whom the feature was assessed; NA, not assessed/not available; PHTS, PTEN hamartoma tumour syndrome.

⁎ Oral features consisted of gingival hypertrophy, high palate, and oral papillomas regardless of location (tongue or mucosa).

All patients (regardless of age) had at least one of the 3 oral features, and 75% had 2 or 3 of the oral features (95% confidence interval [CI] 67%-82%). The sensitivity of ≥2 oral features was 49% (95% CI 37%-62%) in paediatric and 99% (95% CI 91%-100%) in adult patients. No significant differences in sensitivity were found between ≥1 or ≥2 oral features in paediatric or adult patients. In adults, a combination of all 3 oral features was assessed and yielded a sensitivity of 70% (95%CI 57%-80%) which was significantly lower than the sensitivity of ≥1 oral feature (*P* ≤ .001; Table). All features were statistically different between paediatric and adult patients (*P* ≤ .001).

## Discussion

In this large PHTS cohort, easily assessable oral phenotypic characteristics that emerge in adolescence and early adulthood were evaluated for their potential to serve as “red flags” for earlier recognition of PHTS. The presence of at least 2 of 3 oral features (gingival hypertrophy, oral papillomas and a high palate) was observed in 99% of adult PHTS patients. As individual features, gingival hypertrophy was found in 94%, oral papillomas in 88%, and a high palate in 89% of adult PHTS patients. In comparison, in a survey of 17,235 US adults (aged 17 years or older), trained dentist examiners detected papillomas/warts in 0.75% of the population, fibromas in 0.19%, and gingival hyperplasia in 0.09%.^29^ The 3 oral features, gingival hypertrophy, oral papillomas and a high palate, may therefore be promising markers of PHTS in adults.

Consistent with previous studies in PHTS, the adult cohort included more women than men (59 of 86 patients).^30^^,^^31^ Women are more often diagnosed with PHTS as adults compared to men. Men are more frequently identified during childhood due to macrocephaly and developmental delay, consistent with findings relating to intellectual disability and gender in the general population.^30^^,^^32^

The prevalence of oral features was lower in paediatric than adult patients but gradually increased with age so that in the age group 11 to 20 years, the majority of patients had gingival hypertrophy and/or oral papillomas. Thus, although oral features may not be so frequent in paediatric patients, they might still be helpful in the identification of PHTS in older children and adolescents.

The high prevalence of oral papillomas in this cohort (71% overall) is consistent with other PHTS studies, in which the prevalence ranged from 41% to 86%.^30^^,^^33^^,^^34^ The highest estimate (86%) was based on a cohort with the highest proportion of adults, consistent with our observations of the development of oral papillomas with age. Interestingly, older PHTS patients may present to dentists for aesthetic reasons or due to denture discomfort due to oral papillomas (see, for example,^19^^,^^35^). It has been estimated that as many as 40% of older adults (≥50 years) with PHTS could present to dentists in this way.^36^

In adult PHTS patients, the risk of developing any cancer increases markedly from age 30 onwards.^5^ When scoring 2 out of 3 characteristics including macrocephaly, multinodular goitre, and multiple oral features, every patient in our adult cohort could be identified as being at risk of PHTS by the age of 30 or earlier. This indicates that most adult patients could potentially be identified before the development of cancer.^30^, ^31^, ^32^

Although the presence of at least 2 of the oral features was very frequent in our cohort of adults with PHTS (indicating a high sensitivity), the specificity of these findings is less clear. For a combination including macrocephaly, goitre and multiple oral features, there was an estimated specificity of 99.9% based on published data for these abnormalities in the general population (1% for oral papillomas and 0.1% for gingival hypertrophy).^25^ However, there are other potential causes for these oral abnormalities. For example, multiple oral papilloma-like lesions may be found in multifocal epithelial hyperplasia (Heck's disease,^37^ multiple endocrine neoplasia type 2B,^38^ tuberous sclerosis,^39^ and neurofibromatosis type 1.^40^ Gingival hypertrophy is most commonly associated with drugs (notably, calcium antagonist antihypertensive agents, phenytoin and ciclosporin^41^ but also rarely with leukaemia,^42^^,^^43^ scurvy,^44^ or granulomatous conditions such as sarcoidosis.^45^

Dental professionals play an important role in the diagnosis and management of oral lesions and progressive gingival hypertrophy. During a consultation it is important to distinguish inflammatory gingival swelling, that is characterised by redness, tenderness and bleeding of the gingiva in the presence of plaque and calculus, from non-plaque–related lesions. Plaque-induced lesions and swelling are mainly localised in the marginal gingiva and return to a healthy status after plaque removal. In contrast, non-plaque–related lesions often show no signs of redness or swelling, even in patients with suboptimal oral hygiene, and can affect the entire oral cavity, including tongue and palatal mucosa, and can have a papillomatous growth pattern with a cobble stone appearance.^46^ Non-plaque–related gingival hyperplasia can overgrow the patient's teeth and impair oral hygiene, exacerbating the condition. Dental professionals play an important role in helping patients to maintain good oral health. Management of progressive gingival swelling in general includes participation in a professional hygiene programme to eliminate plaque and improve oral hygiene, regular clinical follow-up, and possible surgical removal of the gingival overgrowth.^47^ However, as PHTS patients develop gingival hypertrophy and oral papillomatosis due to the nature of their tissue overgrowth condition, improved oral hygiene or surgical intervention will not reduce or eradicate the tissue overgrowth.^3^^,^^47^

A high palate may be part of the normal range but is also associated with many syndromes and medical conditions. These include Downs syndrome,^48^ Turners syndrome,^49^ Klinefelters syndrome,^50^ fragile X syndrome,^51^ Retts syndrome,^52^ cerebral palsy,^53^^,^^54^ ciliary dyskinesia syndromes,^54^ Marfans syndrome,^55^ Tourettes,^56^ schizophrenia,^57^ and sleep apnoea in otherwise healthy individuals.^58^ This suggests that the presence of a high palate may not be as specific or predictive of PHTS as the other oral features. However, with the possible exception of schizophrenia and obstructive sleep apnoea, the conditions known to be associated with a high palate are likely to be diagnosed during childhood and known to the patient's dentist. Thus, in the absence of an alternative diagnosis, the presence of a high palate could contribute to the sensitivity and predictive value of the other 2 oral characteristics. Overall, most of the alternative causes for gingival hypertrophy, oral papillomas or a high palate will either be readily apparent (such as anticonvulsant medication in a patient with known tuberous sclerosis) or medically significant conditions that warrant a prompt diagnosis.

Importantly, the presence of 2 or 3 of the oral features of PHTS should not be considered diagnostic of PHTS. It would also not be feasible to refer all subjects with at least 2 out of the 3 oral features for genetic counselling and testing. Instead, the presence of these oral features should alert dentists to contemplate potential causes and to consider alerting the patient and their medical practitioner to the oral findings. Referral for genetic testing can then be considered by the patient's medical practitioner who will have access to additional medical history, physical findings and family history.^59^

The main strength of this study is the inclusion of a large number of patients with PHTS, a rare syndrome which most dental practitioners will not have encountered before. In addition, all patients were examined by one of 2 medical experts, reducing variability in the assessment of phenotypic characteristics. The main limitations are the lack of control data, the fact that patients were not examined by a dentist, and the fact that patients’ age was based on the last day of clinical follow-up rather than the age of first diagnosis of each phenotypic characteristic. Nevertheless, conclusions can still be drawn as the cumulative prevalence by a certain age is relevant to dentists who frequently see their patients, especially children, on a regular basis over many years.

## Conclusions

Oral features including gingival hypertrophy, oral papillomas and a high palate develop during childhood and adolescence and are found in the majority of adults with PHTS. Dental professionals, given their expertise, are well-positioned to recognise these oral manifestations and should be aware that the presence of these non-plaque–induced oral features, require a differential diagnosis that could be related to genetic syndromes, including PHTS. They can alert the patients’ medical practitioner to look for other features of PHTS and to consider referral to a clinical geneticist for further assessment and DNA testing, if appropriate. Timely diagnosis of PHTS allows initiation of cancer surveillance and preventative treatment which could spare the individual (and potentially their relatives) morbidity, and even be life-saving.

## Conflict of interest

None disclosed.
